# NB housing study protocol: investigating the relationship between subsidized housing, mental health, physical health and healthcare use in New Brunswick, Canada

**DOI:** 10.1186/s12889-022-14923-x

**Published:** 2022-12-28

**Authors:** J. Woodhall-Melnik, J. R. Dunn, I. Dweik, C. Monette, E. Nombro, J. Pappas, A. Lamont, D. Dutton, S. Doucet, A. Luke, F. I. Matheson, R. Nisenbaum, V. Stergiopoulos, C. Stewart

**Affiliations:** 1 Department of Social Sciences, University of New Brunswick, 100 Tucker Park, Saint John, New Brunswick, NB E2L 4L5 Canada; 2 Department of Health, Aging and Society, McMaster University, Hamilton, ON Canada; 3 Department of Psychology, University of New Brunswick, Fredericton, Canada; 4 Department of Community Health and Epidemiology, Dalhousie Medicine New Brunswick, Saint John, New Brunswick, Canada; 5 Department of Nursing, University of New Brunswick, Saint John, New Brunswick, Canada; 6 MAP Centre for Urban Health Solutions, St. Michael’s Hospital, Toronto, ON Canada; 7 Dalla Lana School of Public Health, University of Toronto, Toronto, ON Canada; 8 Department of Psychiatry, University of Toronto, Toronto, ON Canada; 9 Canadian Mental Health Association, Toronto, ON Canada; 10 Department of Mathematics and Statistics, University of New Brunswick, Saint John, New Brunswick, Canada

**Keywords:** Subsidized housing, Mental health, Physical health, Healthcare use, Housing affordability, Prospective matched cohort design

## Abstract

**Background:**

Income and housing are pervasive social determinants of health. Subsidized housing is a prominent affordability mechanism in Canada; however, waitlists are lengthy. Subsidized rents should provide greater access to residual income, which may theoretically improve health outcomes. However, little is known about the health of tenants who wait for and receive subsidized housing. This is especially problematic for New Brunswick, a Canadian province with low population density, whose inhabitants experience income inequality, social exclusion, and challenges with healthcare access.

**Methods:**

This study will use a longitudinal, prospective matched cohort design. All 4,750 households on New Brunswick’s subsidized housing wait list will be approached to participate. The survey measures various demographic, social and health indicators at six-month intervals for up to 18 months as they wait for subsidized housing. Those who receive housing will join an intervention group and receive surveys for an additional 18 months post-move date. With consent, participants will have their data linked to a provincial administrative database of medical records.

**Discussion:**

Knowledge of housing and health is sparse in Canada. This study will provide stakeholders with a wealth of health information on a population that is historically under-researched and underserved.

## Strenghts and limitations

• This study uses a strong longitudinal, prospective, matched cohort design to investigate a group of low-income households that has yet to be studied
• Administrative data linking will be used to follow health outcomes, using provincial health data after primary data collection is complete
• All members of the waitlist are invited to participate. Therefore, some self-selection bias may exist. However, this cannot be avoided as participation in the research is voluntary
• Potential for attrition is offset using strategic methods for follow-up, contact information sharing with the Department of Social Development, recording multiple contact methods, and the use of incentives

## Background

Socioeconomic factors are widely accepted as fundamentally linked to health [1–3]. Of these factors, income and housing are two of the most pervasive social determinants of health [4, 5]. The World Health Organization argues for access to stable, affordable, and adequate housing to decrease health inequities [6]. Further, the Universal Declaration of Human Rights recognizes the right to housing as part of the right to an adequate standard of healthy living [7]. Canada’s first National Housing Strategy (2018) aims to remove 530,000 households from housing need, defined as spending 30% or more of income on housing costs [8]. With renewed Federal commitment to affordable housing, it is imperative to investigate the impact of publicly subsidized rental housing, referred to as subsidized housing, on the health of a population that experiences multiple inequities. Although public housing increases affordability, there is limited understanding of the contribution of subsidized housing to health. The primary objectives of this study are to investigate the impact of subsidized housing on 1) mental health; 2) physical health; and 3) health care utilization. The secondary objective of this study is to understand factors related to the wellbeing of renters as they wait for subsidized housing.

Housing and health outcome studies often focus on the built environment [9–11] and rehousing programs for persons with severe mental illness [12–15]. Studies that do investigate relationships between subsidized housing and health focus on jurisdictions outside of Canada [16–18]. To date, any studies that systematically investigate the impact of public housing on healthcare use could not be located.

In cross-sectional studies, housing unaffordability is associated with distress [19, 20], lower self-perceived mental health [16], poor physical health and increased healthcare use (e.g., emergency care, hospitalization, and walk-in clinic use) [21–23]. Increasing housing affordability through subsidized housing, in principle, should improve residents’ mental and physical health and decrease avoidable healthcare use; however, there is no longitudinal or quasi-experimental evidence to determine whether commonly used housing affordability programs, such as publicly subsidized housing, are directly associated with improvements in mental health, physical health and healthcare use outcomes.

Although the link between housing affordability and health is established, recent studies indicate that subsidized housing alone may not contribute to health improvements. For example, research from Australia indicates that multiple transitions into subsidized housing are associated with poorer mental health [24]. These findings suggest that, despite increased affordability, a lack of permanency in subsidized housing could produce negative impacts on mental health. Further, evidence from subsidized housing in Chicago indicates that low perceived neighbourhood and housing quality have negative impacts on physical health, despite increased affordability [25].

Renters in New Brunswick, experience high rates of housing unaffordability [26]. In the last decade, the average rent across New Brunswick has increased approximately 40% [27]. Despite large increases in rents, the average provincial income has only increased by 10.2% [28]. Low income and housing unaffordability are the main contributors to housing instability and episodes of homelessness in Canada, which are associated with poor mental and physical health outcomes and higher use of emergency healthcare services [21–23, 29, 30].

Access to subsidized housing increases residual income, which could positively contribute to mental and physical health and changes in rates of hospitalization, walk-in clinic use, and primary care appointments. However, it is unclear as to whether the subsidies are enough to significantly decrease stress in a population that experiences low-income. Further, the act of moving into subsidized housing may produce stress that may negatively impact health and healthcare use [24]. The present study will fill a significant knowledge gap on the relationship between access to subsidized housing, mental health, physical health, and healthcare use.

### Study objectives

The study objectives are as follows:

1. To determine the impact of publicly subsidized housing on mental health [*Is subsidized housing associated with changes in mental health symptomology?]*
2. To determine the impact of subsidized housing on physical health [*Is subsidized housing associated with changes in physical health?]*
3. To determine pre- and post-move healthcare utilization patterns (hospitalizations, walk-in clinic use, and primary care appointments) in adults who receive subsidized housing *[Does healthcare use change with receipt of subsidized housing?]*
4. To explore physical health, mental health, and healthcare use in low-income adults who are waiting for access to subsidized housing *[What is the prevalence of physical health concerns, mental health concerns, and healthcare use in low-income adults who are waiting for access to subsidized housing in New Brunswick?]*

## Methods

This study will use a longitudinal, prospective matched cohort design. Research advocates for the use of longitudinal studies to better assess the relationship between mental health and subsidized housing [31, 32]. This approach is also useful for understanding physical health and healthcare use, as prospective cohort designs are particularly strong when used to relate an outcome (e.g. mental health, physical health and healthcare use) to an event (e.g. receipt of subsidized housing) [33]. In this case, the study design will allow the research team to associate changes in health to receipt of subsidized housing. Further, any potential cohort effects can be adjusted for by accounting for individual sociodemographic variations within the cohort of housing applicants [33, 34].

### Primary data collection

The sampling frame for this study is all public housing applicants in New Brunswick, which includes approximately 4750 households at the study start date. Each household will receive a letter mailed from the Department of Social Development (DSD), which will provide information about the study, a link to an online survey, an email, and a phone number for the study team. Online participation will be encouraged; however, participants may choose to complete the survey over the phone with a Research Assistant or via mail. New Brunswick is a bilingual province so all study materials will be available in French and English.

Email addresses, mailing addresses, and phone numbers will be recorded during each survey to prevent study attrition. Upon completion of each survey, participants will be mailed or emailed a $10 gift card to Tim Horton’s coffee shop. Their names will also be entered into a draw for one of three $500 VISA gift cards. The draw for the gift cards will take place immediately after data collection concludes.

Study participants will enter the study as control group members while they wait for access to subsidized housing. During this time, participants will be asked to complete a baseline survey which asks questions on demographics, self-reported mental and physical health, and a variety of potentially confounding measures, which are described in detail below. After the baseline survey is complete, control group participants will be provided with shorter follow-up surveys at 6, 12, and 18 months following their initial baseline survey that assess changes to the main outcomes (physical and mental health) and variable factors (e.g., experiences of stigma, residential satisfaction, etc.).

The research team will ask participants for their consent to share their names with the provincial DSD. Those who consent will have their name sent to DSD via WatchDox (www.watchdox.com), which is used by the Provincial government to transfer confidential information. Program staff with DSD will check the names provided against offers for subsidized housing each month and will provide the research team with updated information and move dates for those who become housed during the study period. Not all participants will consent to sharing their names; therefore, each survey administered to the control group after baseline will ask participants if they have received subsidized housing. Participants who indicate that they have received subsidized housing will be asked when they moved or started to receive a subsidy and will be moved to the intervention group.

The intervention group will receive additional follow-up surveys at six, 12, and 18 months after they begin receiving subsidized housing. Participants who are not subsidized within 24 months of their baseline participation date will not crossover into the intervention group and their study participation will be complete. At the start of the study, many of the households will have already been on the waitlist for months. Therefore, households at the top of the waitlist or those who experience conditions that assign them priority status (e.g. homelessness or intimate partner violence) will move into housing faster than others. Recruiting from the entire waitlist will ensure that households from the top, middle, and bottom of the waitlist are contacted for study participation.

It is possible that control group participants may remove their names from the waitlist during the study period. If this happens, the previous data collected from these participants will be kept and their study participation will be complete. It is also possible that participants in the intervention group may receive and then lose or leave subsidized housing. If this happens, the research team will note this, and their study participation will be complete. Their data prior to exiting subsidized housing will be included in analyses. Should a large enough portion of participants leave the wait list or subsidized housing, their data will be compared with others who either stayed on the wait list or continued to receive subsidized housing to see if any significant differences exist between the groups.

In the absence of any data reporting CESD-10 findings and data from the DAD in intervention studies similar to ours, we will estimate the power to compare pre- vs post-intervention CESD-10 total scores and healthcare use at the end of the study, using Cohen’s d effect sizes for paired samples [35]. Assuming that there will be 30% attrition by the end of the study, a sample size of 1,138 data pairs achieves 100% power to detect effect sizes ranging from 0.3 (moderate effect size) to 0.8 (large) with a significance level equal to 0.05 using a two-sided paired t-test. As analyses will compare intervention and control periods, the researchers expect that the high power calculated using the paired t-test at the end of the study will approximately hold when we fit mixed models to the data.

### Administrative data linking

This study also uses administrative dataset linking to measure differences in physical and mental healthcare use between the intervention and control groups. With each participant’s consent at baseline, their name and date of birth will be used to link their survey results with their matched records in the New Brunswick Institute for Research Data and Training (NB-IRDT) database. The NB-IRDT is an organization that houses and links data with large, provincial administrative databases. It provides individual level data on education, health, social services use, and employment. The primary data collected through this study will be linked with participants’ healthcare use data from the Discharge Abstract Database (DAD), which provides information on patient billing for hospitalizations, walk-in clinic use, and primary care appointments. The research team will use the date that housing subsidies were received to create a time variable that indicates their receipt of the intervention. The DAD and the time variable will then be used to compare individuals’ hospitalizations, walk-in clinic use, and primary care appointments in the 18 months prior to and following their moves into housing. The same analyses will be performed for individuals in the control group to assess differences between the two groups.

### Scales and measures

The measures proposed for this survey are discussed below. Additional questions may be added into follow-up surveys if deemed necessary by the research team.

#### Primary outcome measures

The primary outcomes for this study are mental health, physical health and healthcare use. In this study mental health is conceptualized as the presence or absence of depressive, anxious, and distress symptoms. Depressive symptomology will be measured using the Centre for Epidemiological Studies Depression Scale Short Form (CESD-10) [36–38]. The CESD-10 is an abbreviated, validated version of the CESD-R. A scoring algorithm is applied to each of the 10 questions and the values from all the questions are summed to provide a score ranging from 0–30, with 10 points on the scale being the clinical cutoff that is used to indicate the presence of depression. However, the scores are also suitable for use as a continuous variable [39, 40]. The Kessler 6 (K-6) will be used to measure distress and anxious symptomatology. The K-6 was designed for the U.S. National Health Interview Survey and measures the presence of distress and anxious symptoms using a simple six item scale [41]. The K-6 is an abbreviated version of the K-10. It is quickly administered and is deemed highly reliable and valid [42–44].

Participants will be asked if they have ever received a mental health diagnosis and will be provided with a list of common psychiatric conditions from which to choose. An option to specify a condition that is not listed will be provided.

To assess physical health, the EQ-5D-5L and EQ-VAS will be administered. The EQ-5D-5L is validated measure comprised of five dimensions of health that relate to quality of life. The EQ-VAS is a visual analog scale to measure reported overall health [45, 46]. Participants will also be asked to self-report any intellectual, developmental, or physical disabilities.

The DAD, which captures physician billing data on hospitalizations, walk-in clinic use, and primary care appointments, will be used to measure healthcare use. The NB-IRDT has yet to receive data on Emergency Department use, so this measure will not be included in the present study; however, once these data are available, a secondary analysis of Emergency Department use may be conducted.

#### Demographic and potential confounding variables

Standard demographic information will be collected from each participant (e.g. gender/sexual identity, income, sources of income, work status, marital status, ethnicity, citizenship status, rural or urban residency, and household composition). The NB-IRDT will provide linked data from the Citizen Registry and Vital Stats, which will allow the researchers to account for chronic and comorbid conditions, and movement out of province or death.

New Brunswick’s DSD has indicated that their subsidized housing tenants often feel stigmatized, and this negatively impacts their experiences of mental health and wellbeing. Although there is no current data to confirm this, recent studies from other jurisdictions suggest that public housing tenants experience perceived or actual stigma which negatively impacts wellbeing [47–49]. To measure stigma, the Self-Stigma Short (SSS) will be administered. This is a 9-item validated scale, typically used to measure stigma of mental illness; however, it allows researchers to replace the condition of interest to meet their own research needs [50]. For the purpose of this study, mental illness will be replaced with public housing applicant (control) and public housing resident (intervention). This will allow the research team to assess whether stigma contributes to mental health in the intervention and control groups.

Data on substance consumption will be collected using six adapted measures selected from the Canadian Tobacco and Drugs Survey [51]. These questions will measure the frequency of alcohol, tobacco, and cannabis consumption over the six-month period preceding each survey. The research team only tracked use of legal substances, as illicit drug use is often associated with secrecy and stigma and the use of illicit substances was not critical to the study [52]. This will allow the research team to control for the impacts of any potential changes in substance use on mental and physical wellbeing.

Social support will be measured using the Oslo Social Support Scale (OSS-3). This scale was selected as it is widely used with a variety of populations; further, it is a brief measure of social support which is important to reduce participant fatigue [53]. The scale consists of three questions which are designed to measure the level of social support that people perceive they have. We will include this measure as social support is highly correlated with physical and mental health [54–57].

#### Housing and neighbourhood measures

Previous studies indicate that housing and neighbourhood satisfaction and quality contribute to mental health [58–64]. The survey will use an abbreviated version of the Residential Environmental Satisfaction Scale (RESS), which is highly correlated with the total RESS scale (0.96) [65]. This scale measures both housing and neighbourhood satisfaction. Participants will also be asked to indicate their housing type (e.g. detached, high rise apartment, etc.), housing tenure, and the number of individuals who live at their primary residence, as these are found to impact mental health [66]. This will allow the research team to determine if potential changes to health and healthcare use can be attributed to perceptions of living environment rather than just the affordability aspect of subsidized housing.

#### Preliminary data analysis

Random effects regression has the advantage of allowing researchers to explicitly account for within-person changes or unmeasured heterogeneity within individuals across time [67]. Unmeasured heterogeneity can be described as the unmeasured consistencies in individuals that might influence mental health and healthcare use within each wave of data collection. The research team will first explore the longitudinal changes in primary and secondary outcomes using descriptive statistics pre- and post-intervention, as well as spaghetti plots. To take advantage of the longitudinal nature of our data, we will estimate generalized linear mixed effects models that we predict will take the following form:

$$G\left({Y}_{i,t}\right)={X}_{i,t}\beta +{Z}_{i}u+{\epsilon }_{i,t}$$

${Y}_{i,t}$ is our outcome variable (see main and secondary outcomes above) and $G$ is an appropriate link function (i.e. logistic for dichotomous variables and identity for continuous variables). ${X}_{i,t}$ is a vector of variables that we will treat as having fixed effects (β), ${Z}_{i}$ is a vector of variables and their estimated random effects (u), and ${\epsilon }_{i,t}$ is the remaining error ${X}_{i,t}$, which will include variables that can influence mental health or healthcare use and might not be orthogonal to housing status, like time on waitlist, age, etc. We will also explore whether seasonality (month) or interview wave (baseline, six month, 12 month, 18 month) are appropriate to include in our model. ${Z}_{i}$ is a vector of random effects. We will start by including random intercepts in ${Z}_{i}$ and their estimated coefficients (u), designed to consider whether individual-specific factors can influence outcomes over time, and potentially include random-slope estimates for variables (like sex) if our summary statistics indicate important differences by covariates.

We will explore the effects of gender, age, housing status and chronic disease morbidity at study entry, and interactions of selected key variables. Without observing the data, the research team cannot commit to more sophisticated modeling approaches, but we have a flexible estimation strategy that allows us to take advantage of the longitudinal nature of the data. Interim analyses will be performed as data are collected.

#### Study retention

New Brunswick’s DSD will partner with the research team to provide access to the study population, recruitment assistance, and monthly updates on receipt of subsidized housing for participants who consent. Prior to obtaining consent at six months, and for individuals who do not consent to share their name with DSD for monthly updates, a screening tool will be used at regular survey intervals to assess whether a participant has received subsidized housing and should be transferred into the intervention group. DSD is committed to using the results of this study to improve the wellbeing of residents who are waiting for and receiving subsidized housing. This study will provide descriptive information on the wellbeing of those waiting for subsidized housing, which may point to the need for additional health supports.

Using a longitudinal study design is advantageous as it allows us to relate any observed mental and physical health effects to exposure to housing affordability concerns. Further, investigating change over time allows us to determine the impact of housing on mental health, physical health and healthcare use when participants move and as they become more settled in subsidized housing. However, a concern with longitudinal cohort studies is study retention.

Some attrition is expected in a longitudinal cohort study. To reduce attrition, Scott’s Engagement, Verification, Maintenance and Confirmation (EVMC) Protocol will be used [48]. Scott’s use of this protocol resulted in a 95% retention rate in their study of individuals who experience high residential instability. The ECVM Protocol involves training research assistants to properly motivate study participants by informing them of the social benefits of their research participation; collecting and updating contact information; scheduling follow-up surveys at the end of each survey; and providing reminder cards with a number for the participants to call should they need to update their contact information.

The social benefits of study participation will be clearly conveyed to participants by research assistants who administer phone surveys or in text through the electronic and mailed surveys. All participants will be asked to provide a mailing address, email address, and phone number each time they participate. Participants who are unhoused while waiting for public housing will be asked permission to contact them at a shelter, agency, or through another mechanism of their choice. All participants will be reminded at the end of each survey that they will be contacted in approximately six months for their next survey. If contact methods are not up to date at their follow-up dates (e.g. phone number is out of service or email bounce back), a reminder card will be mailed to let them know that it is time for their next survey. This letter will provide the research team’s contact information and a request to contact the study team to update their information. DSD will update contact information monthly for all unreachable participants who agreed to have their information shared for the research.

Participation will be incentivized with a draw at the end of the study and a gift card following each survey, which may motivate some participants to maintain up-to-date contact information. A systematic review of study retention methods finds that offering incentives is an optimal practice to increase study retention [68].

## Discussion

This research study has received Research Ethics Board certification (REB 2020–032) from the University of New Brunswick. Before each survey, participants will be asked to provide electronic (online surveys), written (mail surveys) or verbal (phone surveys) consent. They will be provided with or read a copy of the study information letter. Consent will be collected at each survey interval and consent to participation in the main study is mandatory.

At baseline, participants will be asked to provide consent for the research team to contact them for a qualitative follow-up study in the future. They will also be asked to consent to link their data with the NB-IRDT. At the six month follow-up period, participants will be asked for consent to share their names and addresses with the DSD so they may provide the research team updated information should they receive subsidized housing. Participants may complete the survey if they answer no to any of the optional consents.

### Dissemination

The research team will regularly meet with DSD to discuss survey design, recruitment, data use, findings, dissemination, and recommendations arising from the research. For each round of surveys, a two-page plain language summary sheet with key findings will be produced. These sheets will be housed on the Principal Investigator’s institutional website and provided to participants who request study feedback via mail or email. All deliverables will be available in French and English. Once the data are analyzed, the research team will work in partnership with DSD to develop recommendations and design evidence-based interventions. Peer reviewed publication of study findings will be sought.

The research team will host community meetings to share the results with members of the public. A meeting will be hosted in each of the three largest cities in New Brunswick—Moncton, Saint John and Fredericton. Virtual and conference call options will be offered for those who live in remote areas or are unable to attend in person. DSD will co-host these meetings. The research team and DSD will send email invitations to public housing providers, study participants, persons residing in subsidized housing, members of local, provincial, and federal government, and members of non-profit organizations who focus on housing instability, health, and/or poverty reduction. During these meetings, the study team will provide all attendees with a copy of the community report and the plain language summary sheets. The study team will deliver a presentation on our research findings and ask the attendees to share their thoughts on or reactions to our findings. The research team will ask attendees to provide their email addresses if they wish to join a community of practice to collaborate on any interventions that arise from our findings.

## Data Availability

Not applicable.

## Abbreviations

NBIRDT: New Brunswick Institute for Research Data and Training
DSD: Department of Social Development
DAD: Discharge Abstracts Database
EVCM: Engagement, Verification, Maintenance, and Confirmation
CESD-10/CESD-R: Center for Epidemiologic Studies Depression Scale 10/Revised
K-6: Kessler 6
EQ-5D-5L: European Quality of Life 5 Dimension
EQ-VAS: European Quality of Life Visual Analogue Scale
REB: Research Ethics Board
SSS: Self-Stigma Short
OSS-3: Oslo Social Support Scale
RESS: Residential Environmental Satisfaction Scale
